# The Role of Peripheral Inflammation in Clinical Outcome and Brain Imaging Abnormalities in Psychosis: A Systematic Review

**DOI:** 10.3389/fpsyt.2021.612471

**Published:** 2021-02-19

**Authors:** Melisa Kose, Carmine M. Pariante, Paola Dazzan, Valeria Mondelli

**Affiliations:** ^1^Department of Psychological Medicine, King's College London, Institute of Psychiatry, Psychology and Neuroscience, London, United Kingdom; ^2^National Institute for Health Research Mental Health Biomedical Research Centre, South London and Maudsley National Health Service Foundation Trust and King's College London, London, United Kingdom

**Keywords:** inflammation, neuroimaging, biomarker, psychosis, predictor, treatment response

## Abstract

Promising research investigating the association between inflammatory biomarkers and response to antipsychotic and/or adjunctive therapy, observed by improvement in psychiatric assessment, is emerging. Increased inflammation has been suggested to contribute to higher severity of symptoms/treatment resistance through the effects that this has on brain structure and function. The present systematic review aims to clarify the potential role of peripheral inflammatory markers as predictors of clinical outcomes and their association with neuroimaging markers in patients with psychosis. Systematic searches of the literature using the databases PsychInfo, OVID Medline, and Embase were conducted to collate studies investigating the association of inflammatory biomarkers with clinical outcome in patients with psychotic disorders and studies examining the relationships between inflammatory biomarkers and neuroimaging data. Seventeen studies on predictors of clinical outcome and 14 on associations between neuroimaging data and inflammatory biomarkers in psychosis were identified, and risk of bias was assessed using the Newcastle-Ottawa Scale (NOS). The main inflammatory markers associated with clinical outcome in psychosis were interleukin (IL)-6, IL-10, and C-reactive protein (CRP). High levels of CRP and IL-6 were associated with worse clinical outcome and deterioration of symptoms over time; in contrast, increased levels of IL-10 were associated with greater symptoms improvement. Smaller hippocampal volume and reduced cortical thickness were the main neuroimaging markers associated with increased peripheral inflammation. The heterogeneity across the studies (i.e., treatments strategies, duration) suggests that potential prediction power of inflammatory biomarkers could partially depend on the methodologies, supported by the overall NOS ratings of the studies. Future studies may need to consider whether a combination of these inflammatory and neuroimaging markers could further improve our ability of predicting clinical outcome in patients with psychosis.

## Introduction

Psychotic disorders are severe mental health disorders that cause abnormal thinking and perceptions. Although a good number of patients with psychotic disorders will achieve clinical remission after starting antipsychotic treatment, there remains a considerable number of patients who will continue to present persistent psychotic symptoms despite pharmacological treatment (1). Finding the best approach to predict clinical response to antipsychotic treatment is essential if we want to improve treatment strategies for patients currently not responding to antipsychotic treatment. Inflammatory biomarkers have been recently suggested to hold potential as predictors of clinical outcome in patients with psychotic disorders. At the same time, researchers call for an increasing need for combinations of different biomarkers to improve the predictive ability of biomarkers tested in isolation (2). In this context, a better understanding of the association between inflammatory markers and measures of brain structure and function could help us to identify the best neuroimaging markers that could be used in combination with inflammatory ones to improve the prediction of clinical outcome in patients with psychosis.

The presence of a relationship between increased peripheral inflammation and psychotic disorders is well-established in the literature (3–13). One recent review (8) investigated cytokine alterations in patients with schizophrenia and the association with severity of symptoms. The review concluded that these cytokine alterations are not only occurring more consistently in patients with schizophrenia compared with controls, but also suggests that sub-groups of patients such as drug-free patients, recent onset/first episode psychosis patients, or stable patients may have differing cytokine and inflammatory profiles. All the reviews and meta-analyses conducted so far have, however, not focused specifically on investigating the role of inflammatory markers in predicting clinical outcomes, in terms of response to treatment or prediction of changes in symptoms over time. A thorough review of the studies conducted on this topic so far is paramount to understand whether the use of inflammatory markers could be supported in future research and trials that want to target patients with worse clinical outcomes. In the long term, these could help patients bypass the delay in getting the treatment best tailored for them. The present paper builds on the previous reviewed evidence of increased levels inflammatory markers in psychosis and their association with severity of symptoms and focus on the aspect of prediction of clinical outcomes which has not been specifically reviewed in previous papers.

The main inflammatory markers studied in patients with psychotic disorder include C-reactive protein (CRP) and cytokines analyzed from blood samples. CRP is an acute phase protein synthetized by the liver in response to factors released by macrophages and adipocytes; it plays a role in innate immunity as an early defense system against infections and its levels increase following secretion of interleukin (IL)-6 by macrophages and T cells. Cytokines are small protein produced by a broad range of immune cells, including macrophages, lymphocytes and mast cells and represent important signaling molecules for the regulation of the immune response. Interleukin (IL)-6, IL-1β, tumor necrosis factor (TNF)-α are among the cytokines more frequently found to be elevated in patients with psychosis (7, 8) and are mostly viewed as pro-inflammatory cytokines. Other cytokines such as IL-10 have also been reported to be abnormally regulated in patients with psychosis (8); IL-10 is better known for its anti-inflammatory effect. Increased levels of inflammatory markers or an unbalance between pro- and anti-inflammatory cytokines can potentially affect brain structure and function as mainly shown in models of depression, by affecting neurogenesis, neuroplasticity and glutamate function (14).

Neural changes across various brain regions and structures have been suggested as one of the mechanisms through which elevated peripheral inflammation could contribute to development of psychosis and treatment resistance in patients with psychosis. Some studies have previously reported associations between increased peripheral inflammation and brain structure and function abnormalities in psychosis (15–17) as well as in healthy controls (18, 19) and patients with depression (20–25). The natural progression of this research is to investigate which neural changes are mostly associated with abnormalities in inflammatory response in patients with psychosis to facilitate our understanding of the possible neural pathways between increased inflammation and poor clinical outcome in psychosis.

This systematic review aimed to investigate (1) whether peripheral inflammatory biomarkers, such as CRP and cytokines, predict clinical outcome in patients with psychosis and (2) how neural changes detected by various neuroimaging techniques, such as magnetic resonance imaging (MRI), may be associated with these inflammatory markers.

## Methods

In the present review, two parallel systematic searches of the literature were conducted in accordance to the Preferred Reporting Items for Systematic Reviews and Meta-Analyses (PRISMA) guidelines (Appendix A in Supplementary Material) (26). The search included studies published up to June 2020.

### Inflammatory Markers and Clinical Outcome

To identify relevant studies investigating the efficacy of inflammatory biomarkers in predicting clinical outcome in psychosis, a systematic search across the databases PsychInfo, OVID Medline, and Embase was conducted. This search was performed by using three categories of keywords across the databases—“inflammatory markers,” “psychosis,” and “treatment response.” The database search began with these categories; the keywords [“peripheral biomarker” OR “peripheral marker” OR “inflammation” OR “inflammatory^*^” OR “cytokine” OR “interleukin^*^” OR “IL-6” OR “IL-1” OR “CRP” OR “c reactive protein” OR TNF^*^” OR “tumo^*^ necrosis factor”] AND [“psychosis” OR “psychotic disorder” OR “schizophrenia”] AND [“treatment^*^” OR “treatment response” OR “antipsychotic^*^”] were used. In addition to electronic searches, we manually searched relevant meta-analyses and reference lists of the retrieved articles for eligible studies that may have been missed.

Studies were included if they met the following inclusion criteria: (1) studies focusing on individuals with a diagnosis of psychotic disorder at the time of the assessment, (2) at least one inflammatory biomarker measured at least at one time point, and (3) longitudinal design. Studies were excluded if they met one of the following criteria: (1) comorbidity of malignancy or chronic inflammatory disorder, (2) review articles, (3) meta-analysis, (4) conference abstracts (5) posters, (6) book/chapters, (7) case reports, (8) editorials, and (9) letter to the editor/non-research letter/discussion.

### Inflammatory and Neuroimaging Markers

To identify papers which investigated neuroimaging findings in relation to inflammation in psychosis, a parallel systematic search was conducted. This similarly used three categories of search terms across the same databases—“inflammatory markers,” “psychosis,” and “neuroimaging”—and included the same keywords as before except replacing “treatment^*^” OR “treatment response” OR “antipsychotic^*^” with “neuroimage^*^” OR “brain scan” OR “magnetic resonance imaging” OR “MRI” OR “functional magnetic resonance imaging” “fMRI” OR “resting state” OR “connectivity” OR “neuroimage^*^” OR “positron emission tomography” OR “PET scan” OR “computer tomography” OR “CT scan” OR “single photon emission computer tomography” OR “SPECT” OR “diffusion weighted imaging” OR “diffusion tension imaging.”

Studies were included if they met the following criteria: (1) studies investigating neuroimaging abnormalities in patients with a clinical diagnosis of psychosis at the time of assessment, (2) at least one inflammatory biomarker measured at least at one time point—for consistency between this search and the previous which looked at biomarkers and treatment outcome, and (3) use of at least one neuroimaging technique. Studies were excluded if they met the same exclusion criteria described in section Inflammatory Markers and Clinical Outcome, with the exception that both longitudinal and cross-sectional design studies were included. Positron Emission Tomography (PET) studies which reported results on translocator protein (TSPO) expression, but not TSPO associations with peripheral markers were excluded from the final selection. This process is outlined further with the PRISMA diagram in **Figure 2**.

Key information including sample demographics, study drugs, and covariate adjustments as well as major findings for each selected study were collated.

### Quality Assessment and Risk of Bias

Full text screenings and quality assessments for each of the included papers were conducted by one researcher (MK); these were then verified by a second researcher (VM) and any uncertainties were discussed with the other authors until a decision on whether or not to include the paper in the review was reached.

To assess the quality of each study, we used the Newcastle-Ottawa Scale (NOS) (27) for assessing the quality of non-randomized cohort and case-control studies. The NOS is comprised of eight items covering three domains—selection (including representativeness and source of sample), comparability (including study design and considerations in analysis), and exposure (for cohort studies, the exposure domain is instead the “outcome” domain). Each paper can be assigned a score of 9 stars and was rated in accordance with the guidelines outlined in the Table 1 legend as either “good,” “fair” or “poor.” The criteria used to assess the quality rating to each paper can be found in Appendix B (Supplementary Material).

**Table 1:** Summary of star allocation and final rating calculation in accordance with the Newcastle-Ottawa Quality Assessment Scale criteria used to assess the quality of each study.

**Quality rating**	**Selection**	**Comparability**	**Exposure**	**Calculation method**
Good	3 or 4 stars	1 or 2 stars	2 or 3 stars	Must meet ALL three conditions to gain rating
Fair	2 stars	1 or 2 stars	2 or 3 stars	Must meet ALL three conditions to gain rating
Poor	0 or 1 star	0 stars	0 or 1 stars	Can meet ANY of the three conditions to gain rating

## Results

### Study Inclusion and Quality

**Figure 1:**
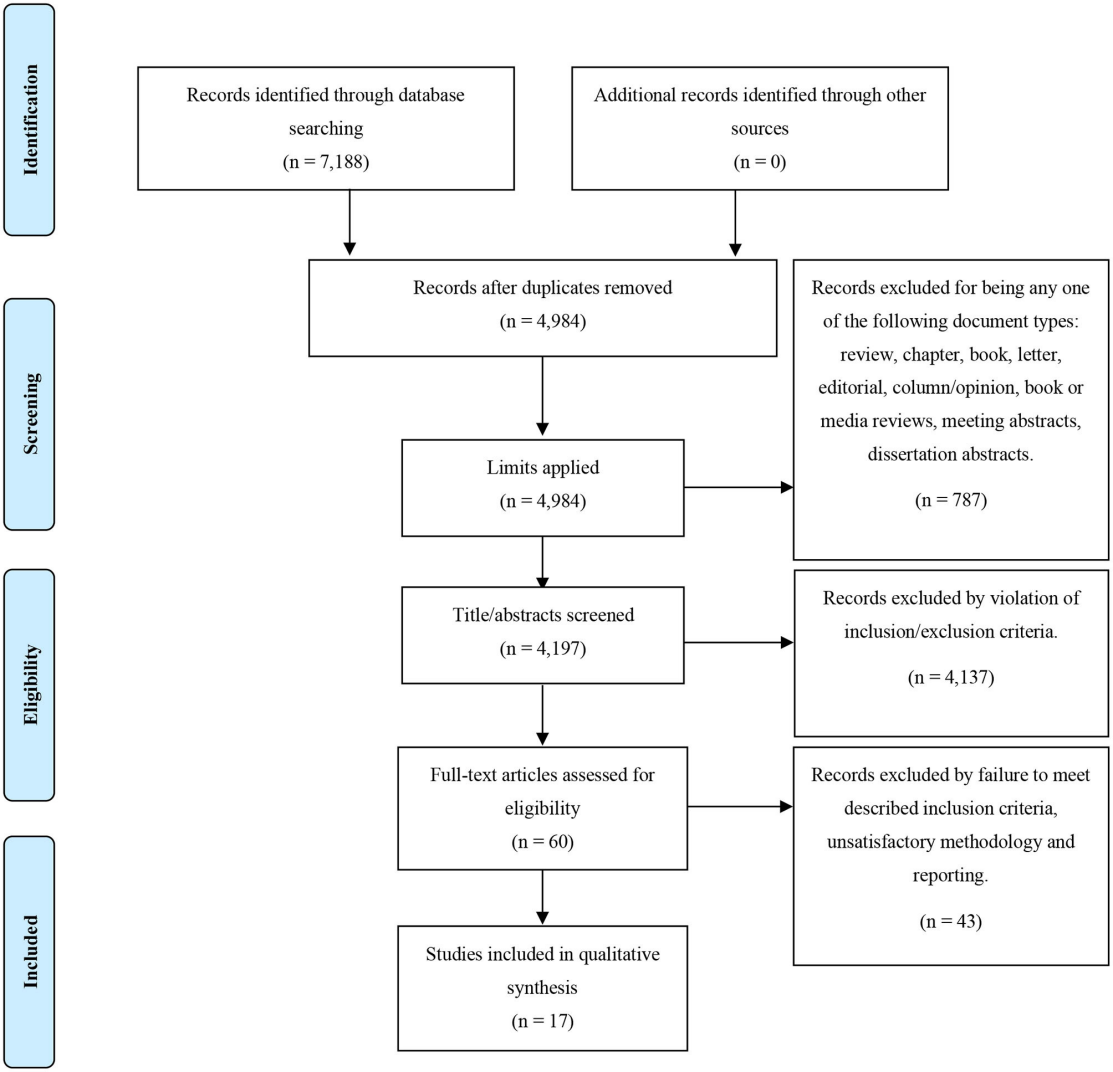
PRISMA flow diagram for our search strategy and the outcome of the inflammatory biomarkers as predictors of clinical outcome in psychosis search.

The literature search of studies investigating inflammatory markers in relation to clinical outcome yielded 7,188 papers which then underwent deduplication. After duplicates were removed, 4,197 papers were identified; following screening of titles to exclude any studies irrelevant to the topic of inflammation in psychotic disorder, 60 papers underwent full-text screenings. Out of these 60, 17 papers met inclusion criteria and were included in the review. This process is demonstrated in full, with the PRISMA flow diagram, in Figure 1. Demographics of the participants from the 17 studies and information on covariates included in the studies' analyses are presented in Table 2.

**Table 2:** Demographics of participants from the 17 studies investigating inflammatory biomarkers as predictors of clinical outcome in psychosis.

**Study**	**Total sample**	**Cases (%women)**	**Controls (%women)**	**Mean age of cases (SD)**	**Mean age of controls (SD)**	**Country**	**Drug**	**Dose**	**Comorbidities**	**Covariate adjustments**
Borovcanin et al. (28)	82^*^	47 (60%)	35 (0%)	35.84 (1.75)	35.46 (1.78)	Serbia	Various	Various	None	Age, sex
Chen et al. (29)	195	95 (40%)	100 (45%)	30.37 (0.87)	31.55 (0.66)	China	RISP + DXM [OR] RISP + Placebo	DXM 60 mg/day	Smoking	Age, sex
Crespo-Facorro et al. (30)	84	56 (35.7%)	28 (57.1%)	26.6 (6.6)	27.1 (3.7)	Spain	RISP, OLZ, HAL	RISP 3–6 mg/day; OLZ 5–20 mg/day; HAL 3–9 mg/day	Smoking	Age, sex
Ding et al. (31)	91	ES 28 (53.6%) Placebo 26 (46.2%)	29 (51.7%)	ES 42.4 (12.7) Placebo 49.7 (9.4)	41.8 (8.5)	China	Escitalopram, Placebo	5 mg/day for the first 3 days, 10 mg/day for day 4 to week 4, and 20 mg/day for weeks 5–8	None	None mentioned
Fathian et al. (5)	208	208 (32.2%)	–	33.5 (13.1)	–	Norway	Various	Various	None	Metabolic syndrome, smoking, being medication naïve, illicit drug use, and educational level
Gonzalez-Blanco et al. (32)	50	50 (38%)	–	30.8 (7.1)	–	Spain	Various	Various	None	Age, sex, smoking, BMI, duration of illness, antipsychotic doses
Hatziagelaki et al. (33)	14	14 (50%)	–	26.5 (6.02)	–	Greece	OLZ	15 mg (*n* = 6) or 20 mg (*n* = 8)	None	None mentioned
He et al. (34)	71	35 (43%)	36 (54%)	26.1 (9.7)	25.8 (5.1)	China	OLZ equivalents	8.19 mg/day (SD = 7.73)	None	Age, sex, BMI, dose
Luo et al. (35)	148	68 (57%)	80 (19%)	34.29 (11.17)	26.77 (5.37)	China	Various	Various	None	Age, sex, alcohol use, smoking
Mondelli et al. (36)	79	49 (32.7%)	30 (36.7%)	28.2 (0.9)	27 (0.8)	United Kingdom	Various	Various	None	Childhood trauma, number of recent stressors, perceived stress
Nettis et al. (37)	88	42 (40.5%)	46 (60.9%)	30.3 (9.8)	28.7 (9.2)	United Kingdom	Various	Various	None	Sociodemographics, BMI, triglycerides
Noto et al. (38)	53	31 (38.7%)	22 (40.9%)	25.8 (6.4)	25.0 (6.7)	Brazil	RISP	Various	None	Age, sex
Sobis et al. (39)	17	17 (41.2%)	–	51.1 (11.8)	–	Poland	ARI	Various	Type 2 diabetes, hypertension (*n* = 1); Arrhythmia (*n* = 1); Smoking (*n* = 14)	Age, BMI, duration of illness, weight, waist circumference
Strzelecki et al. (40)	60	Sarcosine 30 (33.3%) Placebo 30 (50%)	–	Sarcosine 36.9 (11.4) Placebo 40.2 (10.1)	–	Poland	Various + sarcosine	Various	None	Age, sex, smoking, BMI and body fat
de Witte et al. (41)	530	180 (34.4%)	350 (41.1%)	30 (10)	32 (10)	Germany	Various	Various	None	Age, sex, smoking, BMI
Zhang et al. (42)	108	RISP 41 (26.8%) HAL 37 (18.9%)	30 (26.7%)	RISP 43.8 (6.4) HAL 43.7 (8.1)	40.4 (10.3)	China	RISP, HAL, Placebo	RISP 6 mg/day; HAL 20 mg/day	None	Age, sex, smoking, duration of illness
Zhang et al. (43)	75	MINO low dose 25 (48%) MINO high dose 25 (52%) Placebo (52%)	-	MINO low dose 33.04 (7.78) MINO high dose 33.24 (6.48) Placebo 33.68 (6.18)	–	China	RISP, MINO	MINO low = 100 mg/day; MINO high = 200 mg/day	None	Baseline PANSS scores

* This sum excludes the 78 first-episode psychosis patients included as a third;

*DXM, Dextromethorphan; RISP, Risperidone; OLZ, Olanzapine; HAL, Haloperidol; ES, Escitalopram; MINO, Minocycline; ARI, Aripiprazole*.

**Figure 2:**
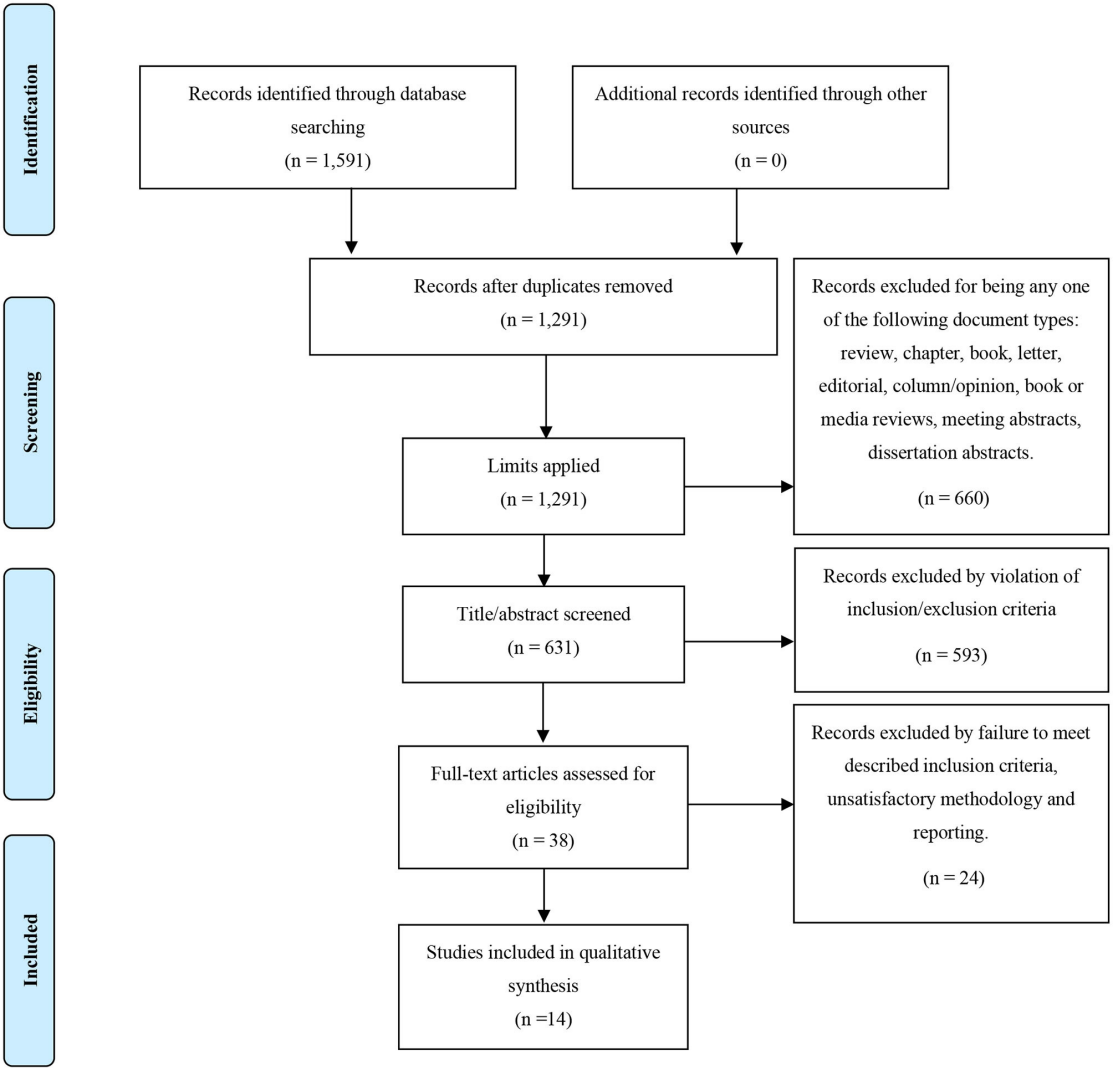
PRISMA flow diagram for our search strategy and the outcome of the inflammatory biomarkers and neuroimaging in psychosis search.

The literature search of studies investigating inflammatory markers in relation to neuroimaging markers yielded a total of 1,591 papers. After applying our defined limits, 631 papers were identified and screened by title and abstract to remove irrelevant papers; the remaining 38 studies underwent full-text screenings. Out of these 38, 14 papers met inclusion criteria and were included in the review. This process is demonstrated in full, with the PRISMA flow diagram, in Figure 2. Demographics of the participants from the 14 studies are presented in Table 3.

**Table 3:** Demographics of participants from the 14 studies investigating associations between inflammatory biomarkers and neuroimaging abnormalities in psychosis.

**Study**	**Total sample**	**Cases (%women)**	**Controls (%women)**	**Mean age of cases (SD)**	**Mean age of controls (SD)**	**Country of participants**
Bossù et al. (44)	100	71 (37%)	29 (31%)	41.8 (11.6)	41.9 (11.6)	Italy
Cannon et al. (45)	170	35 (29%)	135 (46%)	18.8 (3.8)	20.5 (4.6)	United States
De Picker et al. (46)	31	14 (0%)	17 (0%)	32.2 (8.3)	27.2 (5.5)	Belgium
Fillman et al. (47)	86	43 (41.9%)	43 (51.2%)	33.6 (range = 20–48)	32.5 (range = 22–48)	Australia
Hoseth et al. (48)	345	109 (45.9)	236 (55.9%)	30 (13)	35 (17)	Norway
Jacomb et al. (49)	156	85 (40%)	71 (52.1%)	35.8 (8.6)	32.2 (8.3)	Australia
(50)	506	250 (49.6%)	256 (49.2%)	42.5 (14)	41.8 (12.7)	Japan
Lesh et al. (51)	122	69 (12%)	53 (36%)	19.9 (3.5)	19.5 (3.3)	United States
Lizano et al. (52)	887	554 (51.8%)	333 (55.3%)	35.5 (12.5)	37.0 (12.4)	United States
Miller et al. (15)	97	31 (39%)	66 (39%)	34 (0.7) 43 (0.6)	34 (0.7) 43 (0.6)	Finland
Mondelli et al. (16)	79	49 (32.7%)	30 (36.7%)	28.2 (0.9)	27 (0.8)	United Kingdom
Prasad et al. (17)	68^**^	IL-6 33 (45.5%) CRP 37 (43.2%)	IL-6 23 (69.6%) CRP 27 (66.7%)	IL-6 26.39 (8.57) CRP 26.49 (8.21)	IL-6 25.44 (5.50) CRP 26.68 (6.41)	United States
Tsai et al. (53)	32	32 (71.8%)	–	58.8 (7.3)	–	Taiwan
Wu et al. (54)	88	44 (40.9%)	44 (54.5%)	31.25 (11.09)	34.32 (8.23)	China

** *Not all recruited participants were included in the IL-6 or CRP analyses, hence the inconsistency between total, IL-6, and CRP sample sizes*.

Regarding quality of papers, from the total of 31 studies from both literature searches, 17 were rated “good” (10 from the clinical outcome search and seven from the neuroimaging search), five were rated “fair” (all of which were from the clinical outcome search) and 9 were rated poor (six for the clinical outcome search and one from the neuroimaging search). Full results for the risk of bias quality assessment can be found in Table 4 for case-control studies and Table 5 for cohort studies.

**Table 4:** Results of the systematic quality assessment in accordance to the Newcastle-Ottawa Scale for case-control studies.

**Study ID**	**Selection**					**Comparability**		**Exposure**				
	**Case definition adequate?**	**Representativeness of the cases**	**Selection of controls**	**Definition of controls**	**SCORE**	**Matched in design?**	**Score**	**Ascertainment of exposure**	**Same method for both?**	**Same Non-response rate?**	**Score**	**Adequacy**
**Studies investigating inflammatory biomarkers as predictors of treatment outcome in psychosis**												
Borovcanin et al. (28)	Yes*	No	No	No	1	Yes^**^	2	Yes^*^	No	No	1	Poor
Chen et al. (29)	Yes^*^	Yes^*^	No	No	2	Yes^**^	2	Yes^*^	Yes^*^	No	2	Fair
Crespo-Facorro et al. (30)	Yes^*^	Yes^*^	Yes^*^	Yes^*^	4	Yes^**^	2	Yes^*^	Yes^*^	No	2	Good
Ding et al. (31)	Yes^*^	Yes^*^	No	Yes^*^	3	No	0	Yes^*^	Yes^*^	No	2	Poor
He et al. (34)	Yes^*^	Yes^*^	Yes^*^	Yes^*^	4	Yes^**^	2	Yes^*^	Yes^*^	Yes^*^	3	Good
Luo et al. (35)	Yes^*^	Yes^*^	Yes^*^	Yes ^*^	4	No	0	Yes^*^	Yes^*^	No	2	Poor
Mondelli et al. (36)	Yes^*^	Yes^*^	Yes^*^	Yes^*^	4	No	0	Yes^*^	Yes^*^	No	2	Poor
Nettis et al. (37)	Yes^*^	Yes^*^	Yes^*^	Yes^*^	4	Yes^*^	1	Yes^*^	Yes^*^	No	2	Good
Noto et al. (38)	Yes^*^	Yes^*^	Yes^*^	Yes^*^	4	Yes^**^	2	Yes^*^	Yes^*^	No	2	Good
de Witte et al. (41)	Yes^*^	Yes^*^	Yes^*^	Yes^*^	4	Yes^**^	2	Yes^*^	Yes^*^	No	2	Good
Zhang et al. (42)	Yes^*^	Yes^*^	Yes^*^	Yes^*^	4	Yes^**^	2	Yes^*^	Yes^*^	No	2	Good
**Studies investigating associations between inflammatory biomarkers and neuroimaging abnormalities in psychosis**												
Bossù et al. (44)	Yes^*^	Yes^*^	Yes^*^	Yes^*^	4	Yes^**^	2	Yes^*^	No	N/A	1	Poor
Cannon et al. (45)	Yes^*^	Yes^*^	Yes^*^	Yes^*^	4	Yes^*^	1	Yes^*^	Yes^*^	No	2	Good
De Picker et al. (46)	Yes^*^	No	No	Yes^*^	2	Yes^*^	1	Yes^*^	Yes^*^	Yes^*^	3	Fair
Fillman et al. (47)	Yes^*^	Yes^*^	Yes^*^	No	3	Yes^*^	1	Yes^*^	No	N/A	1	Fair
Hoseth et al. (48)	Yes^*^	Yes^*^	Yes^*^	Yes^*^	4	Yes^**^	2	Yes^*^	Yes^*^	N/A	2	Good
Jacomb et al. (49)	Yes^*^	Yes^*^	Yes^*^	Yes^*^	4	Yes^**^	2	Yes^*^	Yes^*^	N/A	2	Good
Kudo et al. (50)	Yes^*^	Yes^*^	Yes^*^	Yes^*^	4	Yes^**^	2	Yes^*^	Yes^*^	N/A	2	Good
Lesh et al. (51)	Yes^*^	No	No	Yes^*^	2	Yes^*^	1	Yes^*^	Yes^*^	N/A	2	Fair
Lizano et al. (52)	Yes^*^	No	No	Yes^*^	2	Yes^**^	2	Yes^*^	Yes^*^	N/A	2	Fair
Miller et al. (15)	Yes^*^	Yes^*^	No	Yes^*^	3	Yes^*^	1	Yes^*^	Yes^*^	Yes^*^	3	Good
Mondelli et al. (16)	Yes^*^	Yes^*^	Yes^*^	Yes^*^	4	Yes^*^	1	Yes^*^	Yes^*^	N/A	2	Good
Prasad et al. (17)	Yes^*^	No	No	No	1	Yes^**^	2	Yes^*^	Yes^*^	N/A	2	Poor
Wu et al. (54)	Yes^*^	Yes^*^	Yes^*^	Yes^*^	4	Yes^**^	2	Yes^*^	Yes^*^	N/A	2	Good

*Scoring criteria (i.e., what terms must be met to earn a star?) and adequacy (i.e., how many stars must be scored in each of the three domains to determine the studies quality?) can be found in Table 3 and Appendix B (Supplementary Material)*.

**Table 5:** Result of the systematic quality assessment in accordance to the Newcastle-Ottawa Scale for cohort studies.

**Study**	**Selection**					**Comparability**		**Outcome**				
	**Representativeness of the cases**	**Selection of non-intervention cohort**	**Ascertainment of exposure**	**Outcome of interest was not present at start**	**SCORE**	**Matched in design?**	**SCORE**	**Assessment of outcome**	**Follow-up long enough for outcomes? (≥2 months)**	**Adequacy of follow-up cohorts**	**Score**	**Adequacy**
**Studies investigating inflammatory biomarkers as predictors of treatment outcome in psychosis**												
Fathian et al. (5)	Yes^*^	N/A	Yes^*^	Yes^*^	3	Yes^*^	1	Yes^*^	Yes^*^ - 6 months	No	2	Good
Gonzalez-Blanco et al. (32)	Yes^*^	N/A	Yes^*^	Yes^*^	3	Yes^**^	2	Yes^*^	Yes^*^ - 1 year	Yes^*^	3	Good
Hatziagelaki et al. (33)	No	N/A	Yes^*^	Yes^*^	3	No	0	Yes^*^	Yes^*^ - 2 months	Yes^*^	3	Poor
Sobis et al. (39)	No	N/A	Yes^*^	Yes^*^	2	Yes^**^	2	Yes^*^	No - 1 month	No	1	Poor
Strzelecki et al. (40)	Yes^*^	Yes^*^	Yes^*^	Yes^*^	4	Yes^**^	2	Yes^*^	Yes^*^ - 6 months	Yes^*^	3	Good
Zhang et al. (43)	Yes^*^	Yes^*^	Yes^*^	Yes^*^	4	Yes^*^	1	Yes^*^	Yes^*^ - 3 months	Yes^*^	3	Good
**Studies investigating associations between inflammatory biomarkers and neuroimaging abnormalities in psychosis**												
Tsai et al. (53)	Yes^*^	N/A	Yes^*^	Yes^*^	3	No	0	Yes^*^	N/A	N/A	1	Poor

*Scoring criteria (i.e., what terms must be met to earn a star?) and adequacy (i.e., how many stars must be scored in each of the three domains to determine the studies quality?) can be found in Table 3 and Appendix B (Supplementary Material)*.

The summarized findings are presented in Table 6 (for the clinical outcome search) and Table 7 (for the neuroimaging search). We will present below separately the results from the studies investigating the association between C-reactive protein and clinical outcome and the results from the studies investigating the association between cytokines and clinical outcome.

**Table 6:** Summary of findings for selected studies investigating inflammatory biomarkers and clinical outcomes.

**Study**	**Treatment**	**Duration**	**Type**	**Biomarkers**	**Assessment**	**Major findings: correlations**	**Major findings: predictors**
Borovcanin et al. (28)	Unspecified	30 days	AAP, TAP	IL-23	PANSS	IL-23: No significant correlation between changes and PANSS scores at baseline nor at 30-day follow-up.	/
Chen et al. (29)	[RISP + DXT] or [RISP + placebo]	11 weeks	AAP, ATVS	IL-1β, TNF-α	PANSS	IL-1β/TNF-α/BDNF ratio: No significant correlations between levels and PANSS scores at 11 weeks.	/
Crespo-Facorro, (30)	RISP, OLZ, HAL	6 weeks	AAP, TAP	IL-12	BPRS, SANS, SAPS	IL-12: No significant correlations between changes in IL-12 levels from baseline to 6-week follow-up and BPRS, SANS nor SAPS scores at 6 weeks.	/
Ding et al. (31)	[OLZ + RISP] or [RISP + ARI] or OLZ or RISP (WITH Escitalopram)	8 weeks	AAP, AD	IL-6 and CRP	PANSS	IL-6: Positive correlation between changes from baseline to 8-week follow-up and changes in PANSS-Negative, PANSS-Total, and PANSS-Cognitive scores. IL-6: Positive correlations between both baseline and 8-week follow-up levels with PANSS-Negative, PANSS-Total, and PANSS-Cognitive scores. CRP: No significant correlation with PANSS scores.	Baseline IL-6 predicted reduction in PANSS-Negative, PANSS-Total, and PANSS-Affective scores.
Fathian et al. (5)	RISP, OLZ, Quetiapine, or Ziprasidone	24 weeks	AAP	CRP	PANSS, CDSS, RBANS	/	Lower baseline CRP predicted cognitive improvement, as measured by the RBANS, following treatment.
Gonzalez-Blanco et al. (32)	Chlorpromazine	Follow-up study	TAP	CRP	PANSS	/	CRP 3–10 mg/L + age predicted both PANSS-Total and PANSS-General scores. CRP 3–10 mg/L + BMI + female sex predicted PANSS–Positive scores.
Hatziagelaki et al. (33)	OLZ	8 weeks	AAP	IL-2, IL-17F, IL-17A, IL-22, IL-1β, IL-21, IL-23, IL-27, IL-4, IL-6, IFN-γ, TNF-α, TGF-β1, TGF-β2, TGF-β3	PANSS	IL-6: Positive correlation between baseline levels and PANSS-Negative score at follow up. IL-6: Positive correlation between baseline levels and % change in PANSS-Negative score from baseline to follow-up. IL-27: Positive correlation between baseline levels and PANSS-Negative score at follow up. IL-27: Positive correlation between baseline levels and % change in PANSS-Negative score from baseline to follow-up.	Higher baseline IL-6 and also higher baseline IL-27 both predicted PANSS-Negative percentage change over time and greater improvement in PANSS-Negative score at 8-week follow-up.
He et al. (34)	Various including RISP, OLZ, HAL, ARI, clozapine, amisulpride	6 months	AAP	IL-1β, IL-4, IL-6, IL-8, IL-12, TNF-α, IFN-γ	PANSS, CGI	IL-6: Positive correlation between baseline levels and PANSS-Negative score.	Higher baseline IL-6 predicted greater improvement in PANSS-Negative score at follow-up. Lower baseline IL-8 predicted greater improvement in PANSS-Negative score at follow-up.
Luo et al. (36)	RISP, ARI, Clozapine Quetiapine OLZ, Ziprasidone,	69 days (±45.66)	AAP	TNF-α, IL-18, IL-6	PANSS	IL-6: Positive correlation between baseline levels and PANSS-Negative scores IL-6: Positive correlations between follow-up levels and PANSS-Positive, PANSS-Negative, and PANSS-Total scores. IL-6: Positive correlations between changes in levels and follow-up PANSS-Positive, PANSS-Negative, and PANSS-Total scores. IL-18: No significant correlation with PANSS scores. TNF-α: No significant correlation with PANSS scores.	/
Mondelli et al. (36)	RISP, OLZ, ARI, Quetiapine	12 weeks	AAP	IL-1β, IL-2, IL-4, IL-6, IL-8, IL-10, TNF-α, IFN-γ	PANSS	IFN-γ: Positive correlation between baseline levels and PANSS-Negative score at follow-up.	Higher IL-6 at baseline predicted poorer clinical outcome at follow-up, as measured by PANSS-Total scores. Higher IFN-γ at baseline predicted poorer clinical outcome at follow-up (PANSS-Total)
Nettis et al. (37)	RISP, OLZ, ARI, HAL, Quetiapine, Zuclopenthixol	52 weeks	AAP, TAP	hsCRP;Factor 1: CRP, BMI, and Triglycerides (TG)	PANSS	Factor 1: Positive correlation with PANSS-Positive, PANSS-Negative, PANSS-Total, and PANSS-Global scores	Factor 1 (CRP, BMI and TG) at baseline predicted 1-year follow-up treatment response, shown by percentage improvement.
Noto et al. (38)	RISP	10 weeks	AAP	IL-1β, IL-10, IL-13, IL-6, IL-7, IL-15, IL-5, IL-12, IL-1RA, IL-2, IL-17, sIL-2R, IL-4, IL-8, IFN-γ, and TNF-α	PANSS	IL-6: Positive correlations between levels and PANSS-Negative, PANSS-Psychosis, PANSS-Affective, and PANSS-Excitation. IL-8: Positive correlations between levels and PANSS-Negative, PANSS-Psychosis, PANSS-Affective, and PANSS-Excitation.	Higher baseline levels of IL-10 and sTNF-R1 predicted greater improvement at 10-week follow-up, as measured by PANSS-Negative and PANSS-Positive scores.
Sobis et al. (39)	ARI	4 weeks	AAP	IL-6, TNF-α, IL-1β, IFN-γ, sTNF-R1, IL-12, IL-23, IL-1Ra, TGF-β1, IL-4, and IL-10 and CRP	PANSS	IL-10: Negative correlations between follow-up levels and follow-up PANSS-Positive, PANSS-Negative and PANSS-Total scores. IL-6/IL-10 ratio: Positive correlation between follow-up levels and follow-up PANSS-Positive, PANSS-Negative, PANSS-Total, and PANSS-Global scores.	/
Strzelecki et al. (40)	[Unspecified Antipsychotic + Sarcosine] or Placebo	6 months	AAP, TAP, GTI	TNF-α	PANSS, CDSS	TNF-α: Positive correlation between levels and CDSS score at baseline. No correlations between TNF-α and any PANSS scores.	Baseline TNF-α did not predict response to sarcosine, as measured by PANSS scores.
de Witte et al. (41)	RISP, OLZ, Quetiapine	6 weeks	AAP	IFN-γ, IL-1α, IL-1RA, IL-5, IL-10, IL-12, IL-15, IL-18, TNF-α	PANSS	IL-10: Positive correlations between changes in levels from baseline to 6-week follow-up and PANSS-Negative, PANSS-Total, and PANSS-General scores. IL-1Ra: No correlations between changes in levels from baseline to 6-week follow-up and any PANSS scores.	/
Zhang et al. (42)	RISP, HAL	12 weeks	AAP, TAP	IL-2, IL-6	PANSS	IL-2: Positive correlation between reduction in levels and reduction in PANSS-Total scores IL-2: Negative correlation between baseline levels and reduction in PANSS-Positive scores.	Lower baseline IL-2 (with lower superoxide dismutase activity and female sex) was associated with greater clinical response, as measured by PANSS scores.
Zhang et al. (43)	RISP, MINO	12 weeks	AAP, TCAB	IL-1β, IL-6, TNF-α	PANSS, SANS	In high-dose minocycline group: IL-6: Positive correlation between reduction in levels and reduction in PANSS-Negative scores. IL-1β: Positive correlation between reduction in levels and reduction in PANSS-Negative scores. In low-dose minocycline group: IL-6: Positive correlation between reduction in levels and reduction in PANSS-Negative scores. IL-1β: Positive correlation between reduction in levels and reduction in SANS scores.	/

*SCZ, schizophrenia; FEP, first episode psychosis; DXM, Dextromethorphan; RISP, Risperidone; OLZ, Olanzapine; HAL, Haloperidol; ES, Escitalopram; MINO, minocycline; ARI, Aripiprazole; AAT, atypical antipsychotic; TAP, typical antipsychotic; ATVS, Antitussives; GTI, glycine transport inhibitor; TCAB, tetracycline antibiotic; CRP, c-reactive protein; TNF-α, tumor necrosis factor-alpha; IL-#, interleukin-#; PANSS, Positive and Negative Syndrome Scale, CDSS, Calgary Depression Scale for Schizophrenia; BPRS, Brief Psychiatric Rating Scale; SANS, Scale for the Assessment of Negative Symptoms; SAPS, Scale for the Assessment of Positive Symptoms; RBANS, Repeatable Battery for the Assessment of Neuropsychological Status*.

**Table 7:** Summary of findings for selected studies investigating inflammatory and neuroimaging markers.

**Study**	**NI**	**Observed region**	**Key biomarkers**	**Major findings**
Bossù et al. (44)	MRI	Hippocampus	IL-18	Lower amounts of free IL-18 were related to smaller hippocampal volume measures in patients with SCZ.
Cannon et al. (45)	MRI	Prefrontal, superior temporal, and parahippocampal regions	TNF-α, IFN-γ, IL-2, IL-10, IL-1RA, CCL2	Rate of prefrontal cortical thinning from baseline to follow-up had significant negative association with higher levels of pro-inflammatory biomarkers – greater in converters (CHR to FEP)
De Picker et al. (46)	PET, MRI	Cerebellum, brainstem, cingulate cortex, thalamus, basal ganglia, amygdala, hippocampus	TSPO, IL-6, IL-8, TNFα, CRP, IL-1RA	Plasma CRP and quinolinic acid were independently associated with lower regional volume of distribution. No significant associations between any peripheral immune markers and TSPO uptake.
illman et al. (47)	MRI	Broca's, Wernicke's	IL-1β, IL-2, IL-6, IL-8 and IL-18	Higher cytokine levels associated with greater reduction in left pars opercularis (Broca's area) volume. IL-1β mRNA levels negatively correlated with left pars opercularis volume
Hoseth et al. (48)	MRI	Hippocampus	Plasma sTNF-R1, osteoprotegerin, IL1-Ra and IL-6	A trend (*p* = 0.09) toward a negative association (b = 0.10) between osteoprotegerin and the volume of the total hippocampal formation after controlling for age, sex, estimated intracranial volume and diagnosis. No significant correlations between any measured cytokines and hippocampal volumes.
Jacomb et al. (49)	MRI	Cortical thickness	CRP	Higher CRP levels were associated with lower cortical thickness. CRP significantly predicted cortical thickness in most regions (frontal pole, medial orbital frontal, lateral orbitofrontal, temporal pole, middle temporal, entorhinal, insula, and paracentral regions).
Kudo et al. (50)	MRI	Hippocampus	Plasma sTNFR2	Higher sTNFR2 levels significantly associated with smaller hippocampal volume in patients with schizophrenia
Lesh et al. (51)	MRI	Total GM and WM	IL-1β, IL-2, IL-4, IL-6, IL-10, IL-12p70, IFN-γ, and TNF-α	Higher IFN-γ levels negatively correlated with whole-brain GM volume. Trend toward a negative correlation between IFN-γ and left middle frontal gyrus thickness. No correlations of cytokines with cortical thickness were observed
Lizano et al. (52)	MRI	Choroid plexus	IL-1b, IL-2, IL-4, IL-6, IL-8, IL-10, IL-12, IL-12p70, IFN- γ, TNF- α, CRP	Higher IL-6 levels associated with larger choroid plexus volume in probands.
Miller et al. (15)	MRI	Hippocampus	CRP, IL-6, IL-17, IL-1α, IL-1β, IL-4, IL-8, IP 10, and MCP1	Higher blood IL-6 levels were a predictor of smaller left and right hippocampal volumes, between ages 34 and 43 years. Significant association between IL-6 levels and hippocampal volumes.
Mondelli et al. (16)	MRI	Hippocampus	BDNF, IL-6, TNF-α	Increased IL-6 expression significantly predicted a smaller left hippocampal volume. IL-6 mRNA levels correlated with left hippocampal volume.
Prasad et al. (17)	DTI	WM and connectivity; Fractional anisotropy and radial diffusivity.	IL-6, CRP	IL-6 levels negatively correlated with fractional anisotropy and positively correlated with radial diffusivity—localized to the forceps major, the inferior longitudinal fasciculus and the inferior fronto-occipital fasciculus. CRP levels negatively correlated with fractional anisotropy within forceps major.
Tsai et al. (53)	MRI	Total GM and WM, hippocampus, amygdala, prefrontal, orbitofrontal, frontal lobe.	Plasma IL1-β, sIL-2R, sIL-6R, IL-1Ra, and sTNF-R1	Higher plasma sTNF-R1 levels significantly associated with lower volume in right amygdala. Higher plasma sIL-2R levels significantly associated with lower volume in the left anterior cingulum. Higher plasma IL-1Ra levels were associated with greater volume of right anterior cingulum
Wu et al. (54)	MRI	Cortical thickness, surface area, and cortical and subcortical GM volumes.	IFN-γ, IL-1β, IL-2, IL-6, IL-8, IL-10, and TGF-β	IL-6 levels significantly associated with the cortical thickness in the left pars opercularis, superior temporal gyrus, right middle temporal gyrus, and pars triangularis. IL-10 levels significantly associated with cortical thickness in right caudal anterior-cingulate cortex and GM volume in frontal gyrus and cingulate cortex. IL-6 levels significantly associated with GM volume in the right lingual gyrusIL-8 levels significantly associated with GM volume in the left medial orbital frontal cortexIL-10 levels significantly associated with GM volume in the left lateral and medial orbital frontal cortex, rostral middle frontal gyrus, right caudal middle frontal gyrus, superior frontal gyrus, isthmus-cingulate cortex, lingual gyrus, and precuneus cortex. None of these were associated with subcortical structures' GM volumes.

*SCZ, schizophrenia; NI, neuroimaging technique; GM, gray matter; WM, white matter; FEP, first episode psychosis; CHR, clinical high risk; AAT, atypical antipsychotic; TAP, typical antipsychotic; TCAB, tetracycline antibiotic; CRP, c-reactive protein; TNF-α, tumor necrosis factor-alpha; IL-#, interleukin-#; PANSS, Positive and Negative Syndrome Scale, BLE, Brief Life Events questionnaire; PSS, Perceived Stress Scale; CECAQ, Childhood Experience of Care and Abuse Questionnaire; SIPS, Structured Interview for Prodromal Syndromes; SOPS, Scale of Prodromal Symptoms; SCID, Structured Clinical Interview for DSM-IV*.

### C-Reactive Protein and Clinical Outcome

Out of the 17 studies identified in the search on clinical outcome, five investigated levels of CRP with treatment outcome and changes in symptoms severity. Clinical outcome was defined by the symptom profile at follow-up or by changes in symptoms severity measured with the PANSS. Duration of follow-up ranged across the studies from 4 weeks (39) to 52 weeks (37). All five of the studies investigated patients on antipsychotic medication and 1 of these studies tested the effects of add-on antidepressant medication (4).

Three of the five studies (5, 32, 37) identified significant associations between baseline CRP levels and changes in symptoms severity and treatment outcome at follow-up. More specifically, González-Blanco et al. (32) found that baseline high levels of CRP (3–10 mg/L) were associated with deterioration in positive, negative, and general symptoms as measured by PANSS at 1-year follow-up (*p* < 0.05), while patients with lower levels of CRP (≤ 3 mg/L) showed improvements in psychotic symptoms across these same domains (*p* < 0.05). Furthermore, in conjunction with age, high levels of CRP predicted total and general symptoms whilst high level CRP in conjunction with Body Mass Index (BMI) and female sex predicted positive symptoms. Similarly, Fathian et al. (5) found that a reduction in CRP levels during the first month of treatment predicted greater cognitive improvement at 6-month follow-up, though they did not investigate correlations with severity of symptoms measured with PANSS. The third study used a different approach by combining baseline levels of CRP with BMI and triglycerides levels in one single immune-metabolic factor. The study showed that this combined metabolic factor predicted poor treatment response, defined as deterioration in overall psychotic symptoms over time, and worse scores of both negative and positive symptoms at 1-year follow-up (37).

Two of the studies examining CRP did not find significant correlations between baseline CRP and later symptoms severity or treatment outcome at follow-up. Both studies focused on a shorter follow-up period, compared with the three positive studies mentioned above. In particular, Ding et al. tested effects of adjunctive treatment with escitalopram over an 8-week period (31) and although they did not find any significant correlation between CRP and PANSS scores, they reported a reduction in CRP levels after 8-weeks of treatment. Sobiś et al. tested the effects of airpiprazole on inflammatory markers over a 4-week period and although they did not find any significant correlation between CRP and PANSS scores, they also identified reduction in CRP over the 4-week treatment (39).

### Cytokines and Clinical Outcome

Out of the 17 studies identified in the search on clinical outcome, 14 investigated levels of cytokines with treatment outcome and changes in symptoms severity. Of note, four of these studies investigated the use of an adjunctive treatment to the prescribed antipsychotic medication; predictors amongst these studies would be predictors of response to add-on minocycline (43), escitalopram (31), sarcosine (40), or dextromethorphan (29) rather than to conventional antipsychotic. The main significant findings were found for the studies testing the effects of add-on minocycline (43) and add-on escitalopram (31) as reported more in detail below.

We found 9 studies investigating IL-6 levels in relation to either clinical outcome or changes in symptoms. Six studies highlight a significant positive correlation between either baseline IL-6 and follow-up PANSS scores or changes in IL-6 and changes in PANSS scores, particularly with PANSS negative symptoms, including the results from two studies with add-on treatment with minocycline or escitalopram (31, 33–35, 38, 43). Four of the 9 studies specifically investigated IL-6 as a predictor of treatment outcome and they all reported baseline IL-6 levels as a successful predictor of treatment response at follow-up (31, 33, 34, 36). Only two studies did not identify a specific correlation between baseline IL-6 levels and changes in symptom severity over 4-week and 12-week trials with aripiprazole or haloperidol/risperidone respectively (39, 42), although the aripiprazole trial reported a reduction in IL-6 levels over the 4-week treatment period (39). Reduction in IL-6 levels from baseline to follow-up were also found to be associated with improvement in cognitive performance and follow-up PANSS positive (*r* = 0.260, *p* < 0.01), negative (*r* = 0.366, *p* < 0.01), and total (*r* = 326, *p* < 0.01) symptoms (35). Similarly, a reduction in in IL-6 levels was associated with an improvement in negative symptoms in patients receiving add-on treatment with a high dose of minocycline (43).

We found seven studies investigating IL-1β levels in relation to either clinical outcome or changes in symptoms. Only one study reported a significant finding, showing correlation between a decrease in IL-1β levels and improvement in negative symptoms only in patients who received add-on minocycline treatment (43).

We found 4 studies investigating IL-2, IL-4, and/or IL-10 levels in relation to either clinical outcome or changes in symptoms. Zhang et al. (42) found that a decrease in IL-2 following antipsychotic treatment significantly correlated with reduction in positive (*r* = 0.38, *p* < 0.01) and total (*r* = −0.36, *p* < 0.01) PANSS sub-score domains. This study also confirmed that higher baseline levels of IL-2 in patients is predictive of poorer response to antipsychotic medication. The other three studies did not find any significant finding with IL-2. No significant finding was reported when looking at the association with IL-4 levels. With regards to IL-10, we found significant findings in three out of four studies. In particular, de Witte et al. showed that IL-10 reduction over time was significantly correlated with improvement in negative (*r* = 0.41, *p* < 0.05), general (*r* = 0.37, *p* < 0.05), and total PANSS scores (*r* = 0.45, *p* < 0.05) after 6-week treatment with antipsychotic (41).

Further, Noto et al. showed that higher baseline levels of IL-10 predicted greater improvement in negative and positive symptoms at 10-week follow-up (38). Finally, Sobiś et al. showed that elevated IL-10 levels were negatively correlated with PANSS-Negative (*p* = 0.03), -Positive (*p* = 0.022), and -Total (*p* = 0.008) sub-scores at 4-week follow-up (39).

We found 5 studies investigating IL-8 and 1 study investigating IL-27 levels in relation to either clinical outcome or changes in symptoms. Lower baseline IL-8 levels predicted greater reduction in PANSS-Negative scores, marking greater improvement in negative symptoms at 6-month follow-up (34). Higher levels of IL-27 at baseline was associated with greater improvement in negative symptoms at 8-week follow-up in a sample of first-episode psychosis patients (33). No other significant finding was reported for these two cytokines in the remaining studies.

Although levels of TNF-α were investigated in 9 studies, no significant association was found between this particular cytokine and clinical outcome or changes in symptoms.

Finally, although six studies investigated IFN-γ levels, only one reported association between this marker and treatment response at 12-week follow-up (36). Other cytokines were investigated in 2 or fewer studies and the results are reported in Table 6.

### Association Between Inflammatory and Neuroimaging Markers

In the neuroimaging studies identified by our search, the most consistently used neuroimaging technique was MRI with 13 studies; one of these studies also included the use of PET imaging. Only one study used Diffusion Tensor Imaging (DTI) as neuroimaging technique. The results are summarized in Table 7.

The hippocampus was the region of interest most commonly studied in association with inflammatory markers (*N* = 8). Increased levels of IL-6 levels were associated with smaller total hippocampal volumes in two studies (15, 16). One study (48) found elevated levels of osteoprotegerin—a tumor necrosis factor receptor—associated with smaller total hippocampal volume, though no correlations with other cytokines, including TNF-α, nor IL-6, were found in the same study. Kudo et al. (50) observed negative association between sTNF-R2 and hippocampal volume while Tsai et al. (53) found sTNF-R1 was positively associated with right amygdala volume. Bossù et al. reported an association between lower levels of pro-inflammatory cytokine IL-18 and smaller hippocampal volumes (44). Fillman et al. reported an association between general elevated inflammation (measured by a specified range of cytokines) and deterioration of Broca's area volume; further, IL-1β mRNA were negatively correlated with volume of this region (47).

Several studies also investigated associations between levels of inflammatory cytokines and cortical thickness. Jacomb et al. (49) reported significant negative association between CRP levels and cortical thickness, and CRP was a reliable predictor of cortical thickness in a wide range of brain regions. Similar findings were reported in a later study (54) in which increased levels of both IL-6 and IL-10 were shown as significantly associated with cortical thickness in a range of brain regions. In contrast, no such correlations were observed by Lesh et al. (51) between CRP, nor with any cytokines, and cortical thickness. Finally, in a study on clinical high risk for psychosis and first episode psychosis patients (45), higher levels of pro-inflammatory markers were negatively associated with rates of cortical thinning between baseline and 12-month follow-up.

We found only 1 DTI study which analyzed the association between peripheral inflammatory markers and brain connectivity, showing a negative correlation between IL-6 and CRP levels with fractional anisotropy within the forceps major (17).

IL-6 was the marker most frequently investigated in association with neuroimaging markers (*N* = 9, plus one study looking at sIL-6R); five of these found significant associations between levels of IL-6 and differences in brain structures, including the two studies finding association with hippocampal volume (15, 16), the study finding association with cortical thickness (54) and the study showing association with fractional anisotropy in the forceps major (17). The fifth study reporting significant association between neuroimaging markers and IL-6 found an association between elevated levels of IL-6 and larger choroid plexus volume (52). Further details of the studies and their findings can be found in Table 7.

## Discussion

To our knowledge, this is the first comprehensive systematic review of the literature investigating the potential role of inflammatory biomarkers as predictors of clinical outcome and their association with neuroimaging markers in patients with psychosis. The following key findings were identified: (1) IL-6, IL-10, and CRP were most consistently correlated with clinical outcome, either as predictors of treatment response or associated with changes in symptoms severity over time, and (2) increased inflammatory markers were mainly associated with smaller hippocampal volumes and reduced cortical thickness in patients with psychosis.

Rather limited research investigating the relationship between various inflammatory biomarkers and treatment efficacy has been conducted (5, 10–12, 55), and thus, the present systematic review highlighted key areas that future research in the field may investigate, including which biomarkers may be most useful to observe and which methodologies appear to work best.

CRP is well-established in the literature as an efficient biomarker of inflammatory state (5, 12, 31, 32). Three studies indicated that decreasing levels of CRP between baseline and follow-up were significantly correlated with improvements in symptom severity following treatment (5, 32, 37). These studies were also the same ones to highlight the predictive power of baseline CRP levels on treatment outcome at follow-up. Of note, these studies had significantly longer durations, with the shortest lasting 24 weeks between baseline and follow-up (5). In contrast, the two other CRP studies (31, 39) which did not show an association of CRP with clinical outcome had signicantly shorter time frames (8 weeks and 4 weeks respectively). This could potentiall suggest CRP as a better marker for long term follow-ups, while other inflammatory markers (such as IL-6) may better suited for shorter term follow-up (1–3 months). Furthermore, of the two studies which did not yield significant correlation between CRP and symptoms severity, Ding et al. (31) investigated the use of adjunctive antidepressant escitalopram, so potentially suggesting that CRP levels are indicative for change in symptoms or prediction of clinical response to antipsychotic treatment only when used independently of any add-on medication.

When looking at the relationship between cytokines and clinical outcome, we found that higher IL-6 levels were associated with worse clinical outcome or deterioration in symptoms over time. Interestingly, one study testing the effect of add-on anti-inflammatory treatment with minocycline reported that the reduction in IL-6 levels induced by the minocycline treatment was associated with an improvement in negative symptoms (43), suggesting that IL-6 would be a potential marker not only to identify patients with worse clinical outcome but that it could also be used to stratify patients populations for add-on treatment with anti-inflammatory medications. IL-10 was the other cytokine which was mostly associated with clinical outcome. IL-10 is mainly viewed as an anti-inflammatory cytokine. Interestingly, three studies reported an association between high levels of IL-10 and greater symptoms improvement and less severe symptoms at follow-up (38, 39, 41), suggesting that an anti-inflammatory response as mediated by IL-10 pathway may contribute to a treatment-induced recovery in psychosis. Future studies would need to consider the possible balance between pro- and anti-inflammatory pathways (IL-6/IL-10 ratio) and how this could also contribute to clinical outcome.

The mechanisms through which peripheral inflammation could contribute to worse clinical outcome are still partly unclear. However, it has been suggested that peripheral inflammation could potentially lead to immune activation in the Central Nervous System (CNS) and affect neuroplasticity, neurogenesis and influence neurotransmitter signaling including the glutamate pathway (14). Only few studies have investigated levels of cytokines in cerebrospinal fluid showing increased levels of pro-inflammatory cytokines such as IL-6, IL-1β, and IL-8 in the CSF of patients with psychotic disorders (12); however, we could not identify any study on CSF cytokines which looked specifically at their association with clinical outcome in patients with psychosis. Other evidence supporting the presence of central inflammation in patients with psychosis derives mainly from post-mortem studies suggesting the presence of activated microglia, the main cells regulating immune response in the brain (9). Various pathways have been suggested in the peripheral immune-to-brain communication including increased permeability of the blood brain barrier, the lymphatic system, and infiltration of peripheral immune cells, such as macrophages, to the brain (56). Unfortunately, the investigation of neuroinflammation in humans *in vivo* is particularly challenging as we lack neuroimaging techniques and tools to be able to identify the low-grade inflammation that characterizes patients with psychosis. The last decades have seen an increase in PET studies using ligands binding the translocator protein (TSPO) as a potential tool to study microglia activation in patients with psychiatric disorders. However, recent studies cast doubt on the efficacy of TSPO ligands as markers of neuroinflammation in psychiatry (57).

In this context our systematic review on the association between peripheral inflammatory markers and neuroimaging findings in patients with psychosis could potentially open other avenues to identify central markers relevant to the peripheral immune-to-brain communication.

Most of the studies identified in this systematic review consistently demonstrate a significant association between increased peripheral inflammation and smaller hippocampal volume and reduced cortical thickness. The association between elevated levels of IL-6 and larger choroid plexus volume is also an interesting finding as the choroid plexus is a particular brain area where the blood brain barrier is usually less tight and could therefore represent one main structure involved in the communication between peripheral inflammation and the brain (52).

### Quality Assessment and Risk of Bias

The NOS was used to assess the quality of the 31 papers included in the present systematic review (27, 58). In 26 of these studies, participant groups were matched by at least age and in 17 papers samples were matched by age and at least one other factor. The remaining five studies which did not have matched samples raises some concerns about the possibility of confounding variable, however, in 2 of these studies a range of factors such as age, sex, alcohol and smoking (35) and childhood trauma and perceived stress (36) were considered covariate adjustments in analysis. Amongst case-control studies (*N* = 24), studies performed better than anticipated with 13 studies achieving a NOS adequacy rating of “good” compared to 6 with a rating of “poor” and 5 “fair”; amongst cohort studies (*N* = 7), the split was far closer to even with 4 studies achieving a rating of “good” and 3 achieving “poor.” With regards to the 3 domains, most studies performed well by reporting sampling methodologies sufficiently; for most, cases were validated by researchers and clinicians directly with the use of structured interviews and/or reference to medical records. The exposure domain was where many studies performed their worst; specifically, with regards to response-rates and attrition which many studies did not provide any information on. While over half of the papers (*N* = 17) did achieve a NOS rating of “good,” 14 did not and as such the trustworthiness of observations and quality of papers should be scrutinized. However, it should be noted that a majority of the shortcomings in the selection and exposure (or outcome for the cohort studies) domains are as a result of a failure to sufficiently report information rather than deficiencies in methodology. For further details on performances across the three domains, see Table 4 for case-control studies and Table 5 for cohort studies.

### Limitations

The heterogeneity in the methodologies and treatment approaches in the reviewed studies is a limit for the interpretation of findings; with the relatively small sample of papers included, this only further hinders our ability to draw decisive conclusions. However, this literature review aims to present the data as it currently stands comprehensively and concisely, so while this diversity should be highlighted, the consistencies between the studies such as observed inflammatory biomarkers, sample sizes and demographics, and statistical analyses allow us to still draw valuable conclusions and suggestions for future studies and clinical trials. Another potential limitation is represented by the observational design of most studies that implies a reduced reliability to prove causal associations; experimental medicine studies in preclinical models using immune challenges may be better placed to understand causality.

## Conclusion

The findings of this systematic review may be used as guidance for future research which aims to use the most promising inflammatory and neuroimaging markers when investigating prediction of clinical outcome in psychosis. The heterogeneity among the examined studies, in terms of analyzed biomarkers and implemented treatments strategies, indicates that the ability of these inflammatory biomarkers to predict clinical outcome are highly dependent on the combinations of treatments, markers and symptoms that are measured. Levels of IL-6, IL-10, and CRP appeared to be the most promising inflammatory biomarkers for prediction of clinical outcome in patients with psychosis, though further research is needed to establish their validation. Interestingly smaller hippocampal volume and reduced cortical thickness were consistently associated with levels of peripheral inflammation; future studies may need to consider whether a combination of these inflammatory and neuroimaging markers could further improve our ability of predicting clinical outcome in patients with psychosis.

## Author Contributions

MK was responsible for conducting the systematic searches, reviewing the studies for eligibility and drafted the first draft of the manuscript. VM conceived the study, oversaw the systematic searches and contributed to the drafting of the manuscript and to the intepretation of the results. CP and PD contributed intellectually to the interpretation of the results. All authors reviewed and contributed to the final manuscript.

## Conflict of Interest

CP and VM have received research funding from Johnson and Johnson as part of a research program on depression and inflammation. CP has received research funding from the Medical Research Council (UK) and the Wellcome Trust for research on depression and inflammation as part of two large consortia that also include Johnson and Johnson, GSK and Lundbeck. PD has received speaker honoraria from Lundbeck and Janssen. The remaining author declares that the research was conducted in the absence of any commercial or financial relationships that could be construed as a potential conflict of interest.
