# Supplementary material for: The Role of Peripheral Inflammation in Clinical Outcome and Brain Imaging Abnormalities in Psychosis: A Systematic Review
Source: Front Psychiatry. 2021 Feb 19;12:612471. doi: 10.3389/fpsyt.2021.612471 (PMC7933584; doi:10.3389/fpsyt.2021.612471)
Supplement: Supplementary file 2 [file Data_Sheet_2.pdf]

Appendix B. Newcastle Ottawa Scale for the assessment of the quality of a case-control and cohort studies; a total of 9 stars can be awarded.

		Star awarded	Star not awarded
**Case-control studies**			
**Selection**	Is the case definition adequate?	Requires independent validation from researchers with reference to primary records (i.e. medical history or hospital records).	Self-report or no description of how a patient was categorised as a case.
	Representativeness of the cases	Representative of general population of patients with a clinical diagnosis of psychotic disorder. All in defined catchment area, hospital/s, or clinic/s.	Does not meet these requirements or study does not report how case participants were selected.
	Selection of Controls	From the community in the same geographical region	Hospital controls (other patients who do not meet the study inclusion criteria for case group).
	Definition of Controls	No current or historical diagnosis of psychosis.	No information on history provided.
**Compa-rability**	Matched in design?	Can be awarded up to two stars if matched for age and at least one other variable. Age must be one of the factors to be able to score 2 stars.	No
**Exposure**	Ascertainment of exposure	Secure records or directly measured by clinicians and/or researchers with a structured interview.	Self-reported information.
	Same method for both cases and controls?	Yes.	No.
	Same Non-Response rate?	Yes.	No or no information on response rates and attrition were provided.
**Cohort studies**			
**Selection**	Representativeness of the cohort	Representative of general population of patients with a clinical diagnosis of psychotic disorder. All in defined catchment area, hospital/s, or clinic/s.	Does not meet these requirements or study does not report how case participants were selected.
	Selection of non-intervention cohort	From the same defined catchment area as the exposed cohort.	Different setting from the exposed cohort.
	Ascertainment of exposure	Secure records or directly measured by clinicians and/or researchers with a structured interview.	Self-reported information.
	Outcome of interest was not present at start	Yes.	No.
**Compa-rability**	Matched in design?	Can be awarded up to two stars if matched/adjusted for age and at least one other variable. Age must be one of the factors to be able to score 2 stars.	No.
**Outcome**	Assessment of outcome	Secure records or directly measured by clinicians and/or researchers with a structured interview.	Self-reported information or no follow-up conducted.
	Follow-up long enough for outcomes? (≥2 months)	Yes.	Less than 2 months or no follow-up conducted.
	Adequacy of follow-up cohorts	Adjusted for missing data or follow-up was less than 2 months after baseline.	No information on missing data, follow-up, or no follow-up conducted.
